# Tryptophan Metabolism and Gut Microbiota: A Novel Regulatory Axis Integrating the Microbiome, Immunity, and Cancer

**DOI:** 10.3390/metabo13111166

**Published:** 2023-11-20

**Authors:** Yingjian Hou, Jing Li, Shuhuan Ying

**Affiliations:** 1 Target Discovery Center, China Pharmaceutical University, Nanjing 211198, China; houyingjian@cpu.edu.cn; 2 School of Life Science and Technology, China Pharmaceutical University, Nanjing 211198, China; 3 Hunan Key Laboratory for Bioanalysis of Complex Matrix Samples, Changsha 410000, China; 4 Shanghai Bocimed Pharmaceutical Research Co., Ltd., Shanghai 201203, China

**Keywords:** tryptophan, microbiota, immune balance, kynurenine pathway

## Abstract

Tryptophan metabolism and gut microbiota form an integrated regulatory axis that impacts immunity, metabolism, and cancer. This review consolidated current knowledge on the bidirectional interactions between microbial tryptophan processing and the host. We focused on how the gut microbiome controls tryptophan breakdown via the indole, kynurenine, and serotonin pathways. Dysbiosis of the gut microbiota induces disruptions in tryptophan catabolism which contribute to disorders like inflammatory conditions, neuropsychiatric diseases, metabolic syndromes, and cancer. These disruptions affect immune homeostasis, neurotransmission, and gut-brain communication. Elucidating the mechanisms of microbial tryptophan modulation could enable novel therapeutic approaches like psychobiotics and microbiome-targeted dietary interventions. Overall, further research on the microbiota-tryptophan axis has the potential to revolutionize personalized diagnostics and treatments for improving human health.

## 1. Introduction

**Figure 1:**
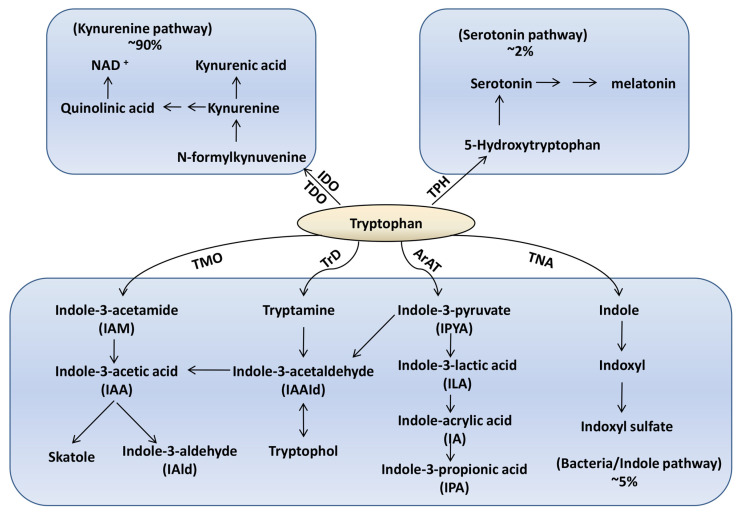
The three major pathways of tryptophan metabolism. IDO: indoleamine 2,3-dioxygenase, TDO: tryptophan 2,3-dioxygenase, TPH: tryptophan hydroxylase, NAD: nicotinamide adenine dinucleotide, TMO: tryptophan 2-Monooxygenase, TrD: tryptophan Decarboxylase, ArAT: aromatic amino acid aminotransferase, TNA: tryptophanase.

Tryptophan is an indispensable and essential amino acid that plays critical physiological roles as a substrate for protein synthesis and its catabolism is an important microenvironmental factor that is involvedin cancer immune cell responses [1,2,3]. Tryptophan (Trp) is metabolized via three major pathways: more than 90% of dietary tryptophan is metabolized through the kynurenine pathway, which generates several active metabolites such as kynurenine (Kyn), kynurenic acid (Kna), 3-hydroxykynurenine (3-OHKyn), 3-hydroxyanthranilic acid (3HAA), and quinolinic acid [4]. This pathway is induced by proinflammatory stimuli and regulated by indoleamine 2,3-dioxygenase (IDO) and tryptophan 2,3-dioxygenase (TDO) enzymes [5,6]. Dysregulation of the kynurenine pathway has been implicated in cancer, neurodegenerative disorders, and psychiatric diseases [7]. Approximately 5% of dietary tryptophan is metabolized through the indole pathwayby the gut microbiota into various indole derivatives, including indole, indole-3-acetic acid (IAA), indole-3-propionic acid (IPA), and others; it primarily occurs in the gut microbiota [8]. The remaining tryptophan is used to synthesize serotonin and melatonin via the serotonin pathway in the gut and brain [9] (Figure 1).

In the past decade, the gut microbiota has emerged as a key regulator of tryptophan metabolism. The colon is home to the densest and most metabolically active community, which comprises more than 10^13^ individual microbial cells [10] and expresses diverse enzymatic activities capable of utilizing tryptophan [11]. Germ-free mice display increased tryptophan levels along with reduced serotonin, indicating the microbial regulation over host tryptophan metabolism [12]. Certain bacterial species like *Clostridium sporogenes* and *Ruminococcusgnavus* have been associated with increased tryptophan catabolism and production of indole metabolites [13]. Conversely, restoringtryptophan levels has been shown to result in the expansion of *Lactobacillus*, which further led to the conversion oftryptophan into intermediate indole-3-lactic acid (ILA) [14,15].

There is accumulating evidence indicating a bidirectional regulatory axis between microbial tryptophan metabolism and the host immune–intestinal system [16]. Alterations along this axis have been associated with cancer, inflammatory bowel disease (IBD), obesity and type 2 diabetes (T2D), chronic kidney disease (CKD), and autism spectrum disorder (ASD) [12,17,18,19]. Elucidating the mechanisms of microbial tryptophan modulation could lead to novel therapeutic approaches such as psychobiotics and microbiome-targeted dietary interventions. This review summarized current literature on the integrated microbiota-tryptophan metabolic axis in health and disease, mechanisms of cross-talk, and implications for human health and medicine.

## 2. Tryptophan-Microbiota Interactions in the Healthy State

### 2.1. Bacterial Species Associated with Tryptophan Metabolism and Metabolite Production

The gut microbiota regulates tryptophan metabolism through microbial enzyme production and conversion into bioactive metabolites [11]. The comparison of conventional mice and germ-free mice showed increased tryptophan availability and reduced kynurenine and serotonin pathway metabolites, indicating microbial catabolism of tryptophan [20,21,22,23]. While the majority of ingested proteins are typically digested and absorbed in the small intestine, it is worth notingthat depending on dietary protein intake, a substantial quantity of proteins and amino acids may transit to the colon [24] where they undergo degradation by a diverse community of commensal bacteria. Specifically, bacterial catabolism of proteins is more pronounced in response to higher dietary protein intake, a reduction in carbohydrate availability within the colon, elevated colonic pH, and an extended transit time through the colon [25,26,27]. This shift towards bacterial proteolytic fermentation is a consequence of the gradual depletion of carbohydrate substrates from the proximal to the distal colon [28]. As a result, the concentration of phenolic compounds, generated through the degradation of aromatic amino acids, is notably more than fourfold higher in the distal colon when compared to the proximal colon [26]. It is important to note that bacterial specialists in proteolysis exhibit lower growth potential compared to generalists and specialists in saccharolysis/lipolysis. This suggests that proteolytic specialists are favored when ecological pressures for rapid bacterial growth are reduced [29]. Nonetheless, it is worth mentioning that the conversion of tryptophan into indole and its derivatives is not exclusive to proteolytic specialists or limited to the distal colon. For example, certain bacterial species such as *Lactobacilli* have been shown to catabolize tryptophan in the stomach and ileum of mice [30]. Numerous bacterial species have been documented to possess the capability to metabolize tryptophan into indole and related derivatives (Table 1).

Indoleamine 2,3 dioxygenase 1 (IDO1) is one of the immune checkpoint blockade genes and is highly expressed in many types of tumor cells; it is the most well studied of the enzymes that initiate tryptophan’s catabolism into kynurenine (Kyn) [59,60]. Tryptophan (Trp) metabolism commits to assisting cancer cells to evade immune surveillance [61,62]. Trp depletion inhibits T cells through the activation of general control non-derepressible protein 2 (GCN2) and down-regulation of the mTORC1 complex [63,64]. In addition, the overexpression of IDO and the accumulation of Kyn in tumor tissue can activate regulatory T (Treg) cells, therefore suppressing the functions of effector T (Teff) cells and natural killer (NK) cells and promoting angiogenesis of the tumor [62,65,66,67,68]. There are many studies that have shown reduced Trp levels and increased Kyn pathway metabolites in CRC patients, indicating increased IDO1 activity [69,70,71]. In addition, Trp metabolism by intestinal microbes may have a role in maintaining immune system stability within the body [72]. Recent research confirmed that intestinal microbes can convert tryptophan into indole and related derivatives. For instance, microbes altered the relative abundance of tryptophan metabolites including indole-3-acetic acid (IAA) and indole-3-acetaldehyde (IAAId) in cecal contents [73,74], which serve as endogenous ligands of the aryl hydrocarbon receptor (AhR). Furthermore, AhR activation promotes the maintenance of ILC3 cells that strengthen the integrity of intestinal mucosa by secreting IL-22 which enhances epithelial barrier function and reduces the number of Treg cells [75]. Thus, the regulation of tryptophan metabolism by intestinal microbes is crucial for host immunity.

Specific bacterial strains have been associated with increased tryptophan catabolism and production of bioactive metabolites. For instance, *Clostridium sporogenes* and *Ruminococcusgnavus* possess tryptophanase and generate indole derivatives such as indole-3-acetic acid (IAA) [13,76]. *Bacteroides thetaiotaomicron* generates indole-3-propionic acid (IPA), which can modulate gut barrier function [77]. *Lactobacillus* spp. can indirectly promote colonic serotonin synthesis by increasing TPH1 expression [78,79,80]. These microbial tryptophan metabolites serve important functions in maintaining intestinal homeostasis. Indole increases the expression of tight junction proteins and mucins to enhance epithelial integrity [81]. IPA promotes intestinal homeostasis by regulating transcript and protein levels of AhR target genes and suppressing cytokine production [82].

### 2.2. Influence of Diet on the Microbiota-Ryptophan Axis

Numerous studies have established associations between specific dietary patterns and the risk of cancer in humans [83,84,85,86]. Consumption of diets characterized by high fiber content, abundant fruits, yogurt, whole grains, extra virgin olive oil, vegetables, and limited intake of animal products has been consistently linked to a reduced risk of cancer [87,88,89,90]. Conversely, a higher risk of cancer has been associated with the consumption of highly processed foods, for instance, diets rich in animal fats and red meat and lower in dietary fiber intake [91,92]. Additionally, dysbiosis in the gut microbiome, as a consequence of a Western dietary pattern, has been correlated with colorectal cancer [93]. Diet plays a central role in shaping the composition of the microbiome, impacting various microbial communities responsible for maintaining physiological homeostasis, modulating immune responses, and facilitating the breakdown of complex polysaccharides [94,95,96]. Therefore, it is important to explore the intricate connections between diet, the microbiome, and cancer. Recent evidence indicates that diet has an apparent effect on both the composition of the intestinal microbes and tryptophan metabolism. High-fat diets have been associated with decreased production of indole and increased levels of kynurenine metabolites by certain bacterial species [97]. Compared to the high-protein-low-fiber diet, the high-fiber-low-protein diet favored the microbial production of indole-3-acetic acid, indole-3-lactic acid, indole-3-aldehyde, and indole-3-propionic acid in both proximal colon and distal colon compartments of the Simulator of the Human Intestinal Microbial Ecosystem (SHIME) [54]. The SHIME is a unique gut model that simulates the entire gastrointestinal tract, including the stomach, small intestine, and various regions of the colon. It is the only in vitro model that combines the entire gastrointestinal transit into one system. Unlike the Reading model [27], the SHIME utilizes peristaltic pumps to connect the different compartments [98]. In addition, skatole (3-methylindole) is a product of bacterial fermentation of tryptophan in the intestine, and its metabolite indole-3-carbinole (I3C) protects wild type mice against intestinal cancer development and reduces hepatic steatosis in mice fed a high-fat diet [99]. In summary, diet powerfully influences the microbiota-tryptophan axis by modulating the composition and metabolic output of the gut microbiome. Further research is elucidating how specific dietary components shape microbial tryptophan processing and production of bioactive catabolites relevant to health and disease.

### 2.3. Effects of Probiotics and Prebiotics on Tryptophan Metabolism

Supplementation with *Lactobacillus* and *Bifidobacterium* probiotics has been demonstrated to boost plasma tryptophan levels, increase serotonin production, and modify tryptophan catabolism in animal models. Specific probiotic strains can reduce inflammation-induced IDO expression, thus preserving tryptophan levels and enhancing its availability [100]. Moreover, specific probiotics and prebiotics have demonstrated beneficial effects on tryptophan metabolism through the kynurenine pathway. For instance, administering *Bifidobacterium* infantisto germ-free mice was able to elevate the Kna levels with no effect on the Kyn concentration and therefore normalized the kynurenine-to-tryptophan ratio in these mice [101]. Prebiotic fructo-oligosaccharides increased the relative abundance of *Lactobacillus* and *Bifidobacterium* and tryptophan levels in the human intestinal tract [102]. Clinical studies have shown that combining probiotics like *Lactobacillus rhamnosusGG* with prebiotics further elevates plasma tryptophan levels compared to each intervention alone [103]. This synergistic effect may be attributed to enhanced growth of tryptophan-producing bacteria [104].

These findings highlight how gut microbes and substrates modulate host tryptophan metabolism. Probiotic bacteria and prebiotic fibers can collectively influence tryptophan availability, catabolism, and the production of bioactive metabolites by promoting the growth and metabolic activities of beneficial gut microbiota. Therefore, understanding the specific mechanisms behind microbial tryptophan utilization and metabolite production is essential for gaining deeper insights into this bidirectional interaction crucial for intestinal health.

## 3. Dysregulation of Gut Microbiota in Disease States

### 3.1. Evidence for Dysbiosis Disrupting the Axis in Cancer, IBD, Mood Disorders, and ASD

It is widely accepted that the dysbiosis of gut microbiota has been associated with disruptions in tryptophan metabolism along the kynurenine and serotonin pathways, contributing to the pathogenesis of several diseases.

Tryptophan can be degraded to generate kynurenine metabolites. The increased expression of indoleamine enzymes IDO/TDO associated with cancer drives tryptophan degradation, resulting in the formation of N-formylkynurenine. This compound is then hydrolyzed by kynurenine formamidase to produce kynurenine. Kynurenine can subsequently follow two pathways: either forming kynurenic acid or undergoing a cascade of enzymatic reactions to yield NAD^+^ [105,106,107]. Previous studies have found that one or more of these enzymes were increased in tumors of the pancreas, breast, and brain [108,109,110]. Previousstudies have demonstrated thattumor-produced kynurenine suppresses cancer immune surveillance. Kyn produced by tumor cells can be exported into the tumor microenvironment, causing T cell inactivation and preventing tumor cell clearance. Kyn can also act as an endogenous ligand for theAhR transcription factor, indicating a cell-autonomous role [111,112,113]. It is believed that AhR activation by tumor-derived Kyn triggers a gene expression program that leads to paracrine immune cell suppression [111]. Additionally, this AhR activation promotes cancer cell proliferation and migration in a cell-autonomous way [114]. Previous studies have shown that the expression of AhR is increased in many cancers, for instance, stomach, liver, prostate, head and neck, breast, brain, and skin cancers [111,115,116,117,118,119,120]. This would suggest thatregulating the expression of AhR plays an important role in tumor aggression.

Colorectal cancer (CRC) is one of the main factors contributing to morbidity and mortality, comprising nearly 12% of all annually diagnosed cancers and cancer-related deaths worldwide [121]. In CRC patients, a depletion of tryptophan metabolism by gut microbes such as *Fusobacteria*, *Enterobacteriaceae*, and *Clostridia* coincides with shunting of tryptophan catabolism toward pro-tumorigenic kynurenine metabolites rather than serotonin synthesis [122]. Recent evidence has demonstrated that *Lactobacillus gallinarum* and its derived ICA could improve anti-PD1 efficacy in CRC by associating with the inhibition of the IDO1/Kyn metabolic circuit as well as the antagonism of Kyn binding to AhR receptors on T cells to inhibit Treg differentiation [123,124,125].

Inflammatory bowel diseases (IBDs) including Crohn’s disease and ulcerative colitis, reveal the significantly differentmetabolic level of Trpbetween healthy individuals and patients. These patients have lower levels of Trp in the serum and feces than healthy subjects [126,127]. Inflammatory bowel disease (IBD) patients exhibit lower concentrations of the AhRagonist IAA in their feces [127]. Interestingly, previous studies have reported an elevated presence of Kyn or higher Kyn/Trp ratios in IBD patients, indicating enhanced tryptophan metabolism through the Kyn pathway during active IBD [128].Additionally, there is an enrichment of Th17 cells and a reduction in Treg cells which can produce anti-inflammatory IL-4 and IL-10, attributed to the interaction of indole metabolites and kynurenine with the AhR on immune cells [77,129]. In general, kynurenine, as an endogenous ligand of AhR, can induce AhR activation when generated in the tumor microenvironment. This function is associated with cancer immunosuppression and sustained activation of AhR, encouraging tumor growth, and affects immune defense [130,131]. However, under inflammatory conditions, AhR activation decreases cytokine production (including TNF, IFNγ, IL-7, IL-12, IL-17, and IL-6) in the intestine, defective AhR activationdetrimentally affects intestinal homeostasis [132]. Appropriate levels of AhR activation are required to maintain intestinal homeostasis. Decreased levels of indole derivatives like IAld can impair intestinal epithelial integrity by inhibiting AhR [30].

In obesity, the relationship between obesity and gut microbiota is a two-way street. Recent studies indicate that the ratio of *Firmicutes*-to-*Bacteroidetes* is not a significant factor in human obesity [133]. It is rather important to focus on distinct bacteria which are associated with obesity such as the family *Christensenellaceae* and the genera *Akkermansia*, *Bifidobacteria*, *Methanobacteriales*, and *Lactobacillus* [134]. The levels of bacterially generated tryptophan metabolites, including indoles, IPA, and indole sulfuric acid (ISA), are diminished in the blood samples of individuals with type 2 diabetes when compared to lean controls. Elevated serum concentrations of IPA have also been linked to a decreased prevalence of T2D [134]. Several indole derivatives resulting from the gut microbiota’s conversion of tryptophan play a role in the development of metabolic syndrome. For instance, IAA-induced IL-35^+^ Breg cells have the potential to influence obesity induced by a high-fat diet [135]. Targeting microbial tryptophan catabolism may support weight management efforts.

Autism spectrum disorder (ASD) has been associated with both microbial depletion of tryptophan via the kynurenine pathway and decreased production of serotonin by select bacteria in the gut [136]. These alterations may relate to pathological changes in behavior and social function. Modulating the gut microbiota through dietary interventions has yielded some improvements in behavioral symptoms in autistic children [137]. Optimizing microbial tryptophan metabolism may support microbiota-gut-brain axis communication and ameliorate autism severity.

In depression and anxiety disorders, previous studies have reported a shift in the composition of gut microbes with the capacity to synthesize serotonin and engage with the gut–brain axis. Reduced levels of *Lactobacillus* and *Bifidobacterium* spp., coupled with an elevated relative abundance of *Alistipes* and *Ruminococcusgenera*, exhibit associations with alterations in tryptophan metabolites along the kynurenine pathway among affected patients [138]. Interventions restoring beneficial serotonin-producing bacteria may hold promise for mood disorders.

### 3.2. Potential Mechanisms of Tryptophan Modulation by Specific Microbes

Gut microbes may modulate tryptophan and its downstream metabolites through several mechanisms. Tryptophancan be transaminated into indole-3-pyruvic acid via an unstable intermediate by the action of tryptophan aminotransferase [139]. In addition to formation from the kynurenine pathway, kynurenic acid can also be formed from indole-3-pyruvic acid via the unstable kynurenic acid intermediate generated with participation of reactive oxygen species (ROS) [140]. In addition, microbiota can stimulate TPH1 activity by its metabolites (e.g., butyrate), directly influencing serotonin and probably melatonin synthesis [141]. The gut microbes play a significant role in tryptophan metabolism, specializing in production of indoles [11]. They metabolize Trp into indole by tryptophanase, into tryptamine by decarboxylase (ALAAD), and into indole-3-pyruvic acid by Trp aminotransferase. The gut microbes amplify the variety of tryptophan catabolites through oxidative and reductive pathways generating various indole derivatives. IPYAcan be converted into other indole derivatives such as ILA, IA, IPA, and also into IAAId, which can then be further processed into indole-3-acetic acid (IAA) and IAld [17]. These compounds are produced by gut microbiota and can be detected in the circulation, feces, and urine [142,143,144]. When absorbed into the circulation, indole can also be transformed into indoxyl sulfate, which has been linked to chronic kidney diseases and cardiovascular issues [145]. However, other indole derivatives such as ILA, IAA, IPA, and IAld play important roles in maintaining intestinal homeostasis, promoting barrier integrity, stimulating epithelial renewal, and regulating mucosal immune responses [30,81,146,147]. Indole is a potent ligand for AhR [148] and is the main bacterial metabolite of tryptophan. It has been shown to have various protective effects in the gastrointestinal tract. These include regulating bacterial motility, promoting antibiotic resistance, inhibiting invasion of host cells by virulent bacteria, ameliorating intestinal inflammation, suppressing the production of proinflammatory chemokines, and increasing the production of anti-inflammatory cytokines [149]. To sum up, indole derivatives are also endogenous ligands of AhR, including tryptamine, skatole, IA, IAA, ILA, IAld, IAAld, and IPA [30,150,151,152,153,154].

Bacterial genes not only metabolize tryptophan-derived metabolites but also provide substrates that can fuel critical host metabolic pathways, such as short-chain fatty acids (SCFAs) [155]. Propionate, butyrate, and acetate make up the specific SCFAs. In addition, the role of SCFAs in protecting against gut inflammation and regulating colonic Treg homeostasis has been well demonstrated [156,157]. For instance, SCFAs decrease STAT1 expression leading to the inhibition of the IFNγ dependent and STAT1-driven transcription of IDO1. In addition, butyrate impairs IDO1 transcription through a second mechanism in a STAT1-independent manner, which may be attributed to its histone deacetylase (HDAC) inhibitory properties [155]. Moreover, butyrate is able to down-regulate IDO1 expression in human intestinal epithelial cells [158]. As we discussed before, the kyn pathway is closely related to the tumor immune escape mechanisms, with the prerequisite for this process being that kynurenic acid is synthesized by IDO1.Thus, microbes can directly and indirectly control tryptophan metabolism through multiple integrated pathways.

### 3.3. Contribution to Pathogenesis of Immune, Metabolic, and Disease Progression

Dysregulation of tryptophan metabolism contributes to disease progression. Activation of the kynurenine pathway leads to immunosuppression, facilitating tumor escape [159]. The induction of TDO2 transcription is driven by proinflammatory cytokines such as IFNγand downstream factors including NF-κB and C/EBPβ [160]. Additionally, activation of the AhR by kynurenine establishes a positive feedback loop, further stimulating IDO1 expression [161]. IDO1 exhibits significant overexpression and is indicative of a poor prognosis in numerous malignancies, with a high IDO1 transcript level serving as a universal adverse prognostic factor in solid tumors. Moreover, heightened IDO1 expression correlates with tumor differentiation, distant metastasis, and an advanced clinical stage [162,163]. Proinflammatory cytokines, such as IFNγ, interleukin-1β (IL1β), and tumor necrosis factor alpha (TNFα), activate IDO1 expression via the JAK/STAT pathway [164,165]. Furthermore, similar to TDO2, a high level of IDO1 transcription in cancer cells is sustained through an AhR-IL6-STAT3-driven positive feedback mechanism [166]. Therefore, dysregulation of tryptophan metabolism can lead to immunosuppression and activate the kynurenine pathway, further resulting in significant overexpression of IDO1, which is associated with a poor prognosis in various malignancies. However, the indole derivativesmetabolized by gut microbiota can regulate the expression of IDO1, which eventually affects the immune response. Overall, disrupted microbial tryptophan metabolism contributes substantially to immune, metabolic, and disease progression.

## 4. Therapeutic Opportunities

### 4.1. Targeting the Microbiota-Tryptophan Axis for Disease Treatment and Prevention

The gut microbiota plays a crucial role in regulating tryptophan metabolism and diverting tryptophan catabolism towards the production of bioactive compounds that influence immunity, metabolism, neurotransmission, and more. This discovery has indicated novel therapeutic opportunities to target the dysregulated microbiota-tryptophan axis for the prevention or treatment of diverse conditions like cancer, inflammatory bowel disease, mood and cognitive disorders, and neurodevelopmental illnesses [22,58,150,167]. Administering certain indole derivatives in animal models has been shown to have positive effects. For instance, IAA administration resulted in ameliorative colitis symptoms [32]. Administration of IAld has anti-inflammatory effects in treating DSS-induced colitis and improving intestinal inflammation caused by bacterial infections [30]. Additionally, it has been found to increase the production of IL-22, further suppressing inflammatory responses [168]. Moreover, the significant effectiveness of indole-3-acetate (I3A) in reducing steatosis and inflammation in mice models highlights its potential as a safe treatment option for non-alcoholic fatty liver disease (NAFLD) [169]. Administration of IPA has been shown to significantly induce expressionof IL-10 receptor protein 1 in cultured intestinal epithelial T84 cells, which further supports the role of IPA in the maintenance of intestinal immunity [82].

A reduction in IFN-γ production during immune activation and a significant increase in plasma tryptophan levels following chronic administration of *Bifidobacteria* were observed in the rat experiment [101]. In addition, previous research showed that serotonin can be produced by *Lactobacillus plantarum* in arginine decarboxylase broth (ADB) [170]. *Lactobacillus rhamnosus GG* has been reported to promote butyrate production, ultimately stimulating TPH1 activity by butyrate and regulating serotonin synthesis [141,171]. In addition, *Lactobacillus amylovorus* and *Lactobacillus plantarum PS128* have been reported to regulate serotonin synthesis as well [79,80]. These probiotics counteract inflammation-induced tryptophan depletion, suggesting therapeutic utility in depression, anxiety, and other disorders associated with dysregulated tryptophan metabolism [100]. Prebiotic fibers like galacto-oligosaccharides that stimulate indigenous *Lactobacillus* and *Bifidobacterium* growth increased SCFAs levels in clinical studies [172], which are the substrates that can mediate tryptophan metabolism in the host. Fecal microbiota transfer from healthy donors modulatedtryptophan and serotonin levels, which indicates microbiota transplantation as another modulatory strategy [78,173]. Potential strategies aim to favorably restructure the gut microbial community and functional capacity to rectify imbalances in tryptophan metabolism through probiotics, prebiotics, fecal microbiota transplantation, and combinatorial therapies. Ongoing investigations aim to identify the most promising probiotic strains, efficacious prebiotics, and optimal microbiota compositional and functional profiles to beneficially impact the microbiota-tryptophan axis across diverse disease contexts.

### 4.2. Combination with Immunotherapies and IDO Inhibitors

Since tryptophan metabolism regulates immune cell responses, modulating the microbiota-tryptophan axis may provide synergistic benefits when combined with cancer immunotherapies. Previous research has revealed that an abundance of gut-resident *Lactobacillus* correlates with IDO1 activity and Th17 cells. Supplementation of SIV-infected macaques with *Lactobacillus* spp. can reduce IDO1 activity and may have the capacity to mitigate the loss of gut barrier-promoting human Th17 cells [174]. Bacterial populations, specifically *Bifidobacterium longum*, *Collinsellaaerofaciens*, and *Enterococcus faecium*, exhibit increased prevalence among individuals who respond favorably to treatment. Conversely, the effectiveness of immune checkpoint blockade therapies is attenuated in the presence of antibiotic administration [175,176,177], which indicates that the combination of certain gut microbes and IDO inhibitors could be a promising treatment. While most current research is focused on the synergistic effects of microbiota and PD-L1 inhibitors, there remains limited literature regarding the concurrent use of IDO1 inhibitors. In fact, IDO inhibitors have led to disappointing results in clinical trials, including epacadostat (INCB024360), BMS-986205, indoximod, navoximod, KHK2455, LY3381916, MK-7162, and NLG802. Notably, clinical trials combining IDO1 inhibitors with other immunotherapies such as PD1 and PD-L1 immune checkpoint inhibitors may be a promising method [178]. However, the combination of microbiota modulation and IDO1 inhibition holds great promise for enhancing the efficacy of immune checkpoint inhibitors. Such combinatorial therapies highlight the potential of targeting dysregulated tryptophan catabolism using the gut microbiota to overcome tumoral immune suppression and improve outcomes across diverse malignancies.

## 5. Challenges and Limitations of Microbiota-Based Therapies

However, several challenges remain in translating insights from the microbiota-tryptophan axis into viable therapeutic interventions. There aremany critical questionsthat need to be addressed, for instance, identifying optimal single or combined bacterial strains and efficacious doses and timing, understanding inter-individual variability in clinical responses and the impact of diet, and elucidating causal mechanisms linking gut microbiome structure to tryptophan processing. Safety, tolerability, long-term effects, production challenges, and regulatory approval also require further study, particularly for combining microbial therapeutics with small molecules. In addition, the temporal sequence and causal relationships between microbiota alterations and tryptophan pathway disruptions need to be precisely delineated and mapped across different disease settings and populations. Despite current limitations, therapeutically manipulating the gut microbiota and tryptophan metabolism holds remarkable promise for modulating immunity, metabolism, neurotransmission, and more.

## 6. Summary

**Figure 2:**
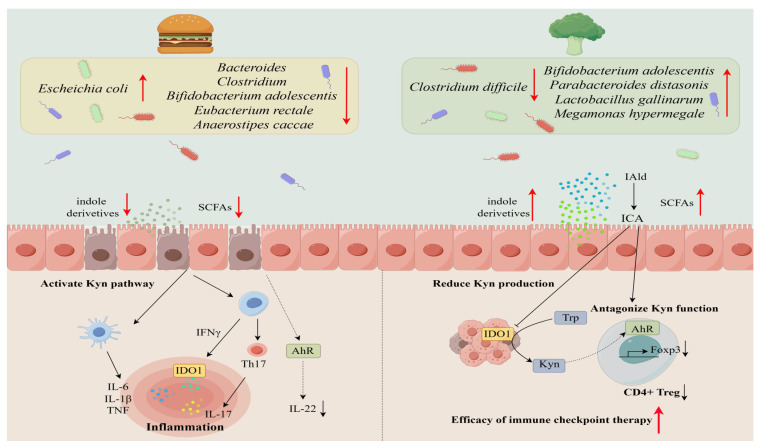
**The impact of different diets on microbiota structure leads to tryptophan metabolism abnormalities**. A high-fat diet reduces the levels of indole derivatives and inactivates AhR attenuated expression of IL-22, ultimately leading to proinflammatory outcomes (**left**); conversely, a high-fiber diet can improve the efficacy of immune checkpoint therapy through specific bacterial strains. Such actions are associated with the IDO1/Kyn metabolic circuit as well as the antagonism of Kyn binding to AhR receptors on T cells to inhibit Treg differentiation (**right**).

In conclusion, the gut microbiota plays a pivotal role in regulating tryptophan metabolism along the kynurenine and serotonin pathways through microbial enzyme production and metabolite generation. Preclinical models and human studies support a regulatory microbiota-tryptophan axis influencing immune function, metabolism, neurotransmission, and disease states. Diet is another key factor influencing the microbiota-tryptophan axis, with high-fat diets decreasing beneficial indole-producing bacteria and increasing tryptophan degradation, while high-fiber diets have the opposite effect. Dysbiosis and disrupted microbial control over tryptophan catabolism contributes to pathogenesis of cancer, IBD, mood disorders, obesity, ASD, and more. The gut microbiota modulates tryptophan availability through direct enzymatic activity as well as production of metabolites like short chain fatty acids that influence host cell tryptophan metabolism (Figure 2). Therapeutic targeting of the microbiota-tryptophan axis through probiotics, prebiotics, and microbiota transplantation shows promise but requires further optimization. Overall, a complex bidirectional relationship exists between gut microbes and host tryptophan metabolism, with intricate effects on physiology and disease. Further research on these interactions may enable novel diagnostics and therapies that harness the microbiota-tryptophan axis to improve human health.

## Figures and Tables

**Table 1:** Microbiota-derived tryptophan metabolites and gut microbiota (↑ represents an increase, ↓ represents a decrease).

Tryptophan Metabolism	Producers	Diet Influence	Impact on Metabolism	Impact on Microbes	Reference
Indole	*Bacteroides thetaiotaomicron*	High-fat diet	Indole Production↓	*Bacteroides* spp.↓ *Escheichia coli*↑ *Clostridium*↓	[11,31,32]
	*Bacteroides ovatus*				
	*Clostridium limosum*				
	*Clostridium bifermentans*				
	*Clostridium malenomenatum*				
	*Clostridium lentoputrescens*				
	*Clostridium tetani*				
	*Clostridium tetanomorphum*				
	*Enterococcus faecalis*				
	*Escheichia coli*				
	*Fusobacterium nucleatum*				
	*Haemophilus influenza*				
	*Proteus vulgaris*				
	*Paracolobactrumcoliforme*				
	*Salmonella enterica*				
	…for more see [33]				
Indole-3-acetic acid (IAA)	*Bacteroides thetaiotaomicron*	High-fat diet	IAA Production↓	*Bifidobacterium* spp.↓ *Bacteroides*↓ *Bifidobacterium adolescentis*↓	[31,34,35,36,37,38]
	*Bacteroides ovatus*				
	*Bacteroides fragilis*				
	*Bifidobacterium adolescentis*				
	*Bifidobacterium pseudolongum*				
	*Clostridium difficile*				
	*Clostridium lituseburense*	High-fiber diet	IAA Production↑	*Bifidobacteriumadolescentis*↑ *Clostridium difficile*↓	
	*Clostridium sporogenes*				
	*Escherichia coli*				
	*Eubacterium hallii*				
	*Eubacterium cylindroides*				
	…for more see [39]				
Indole-3-acrylic acid (IA)	*Clostridium sporogenes*	High-fiber diet	IA production ↑	*Parabacteroides distasonis*↑	[40,41]
	*Peptostreptococcusrussellii*				
	*Peptostreptococcusanaerobius*				
	*Peptostreptococcusstomatis*				
	*Parabacteroides distasonis*				
	…for more see [42]				
Indole-3-propionic acid (IPA)	*Clostridium sporogenes*	High-fat diet	IPA Production↓	*Clostridiumsporogenes*↓	[43,44,45,46,47,48,49,50]
	*Clostridium caloritolerans*				
	*Clostridium botulinum*	High-fiber diet	IPA Production↑	*Clostridium*↑ *Bifidobacterium*↑ *Lactobacillus*↑ *Peptostreptococcus*↑	
	*Peptostreptococcusasaccharolyticus*				
	*Peptostreptococcusrussellii*				
	*PeptostreptococcusanaerobiusCC14N*				
	*Peptostreptococcusstomatis*	Ketogenic diet	IPA Production↓	*Lactobacillus murinus* ↓	
	…for more see [51]				
Indole-3-lactic acid (ILA)	*Anaerostipeshadrus*	High-fat diet	ILA Production↓	*Eubacterium*↓ *Eubacterium rectale*↓ *Anaerostipescaccae*↓ *Bifidobacterium adolescentis*↓	[37,52]
	*Anaerostipescaccae*				
	*Bacteroides thetaiotaomicron*				
	*Bacteroides eggerthii*				
	*Bacteroides ovatus*				
	*Bifidobacterium adolescentis*				
	*Bifidobacterium bifidum*				
	*Bifidobacterium pseudolongum*				
	*Clostridium bartlettii*				
	*Clostridium sporogenes*				
	*Escherichia coli*	High-fiber diet	ILA Production↑	*Lactobacillus*↑ *Megamonas*↑	
	*Eubacterium rectale*				
	*Eubacterium cylindroides*				
	*Faecalibacteriumprausnitzii*				
	*Lactobacillus murinus*				
	*Lactobacillus paracasei*				
	*Lactobacillus reuteri*				
	*Megamonas hypermegale*				
	… for more see [53]				
Indole-3-aldehyde(IAld)	*Lactobacillus johnsonii*	High-fiber diet	IAId Production↑	*Lactobacillus*↑	[54]
	*Lactobacillusreuteri*				
	*Lactobacillusacidophilus*				
	*Lactobacillusgallinarum*				
	… for more see [30]				
Indole-3-acetaldehyde (IAAld)	*Escherichia coli*	/	/	/	[55]
Tryptamine	*Firmicutes C. sporogenes*	High-fat diet	Tryptamine↓	*Bacteroides*↓ *Escheichia coli*↑	[52,56]
	*Clostridium sporogenes*				
	*Escherichia. coli*				
	*Ruminococcusgnavus*				
	*Bacteroides*				
3-methylindole (skatole)	*Bacteroides thetaiotaomicron*	High-fat diet	complex manner	/	[57,58]
	*Butyrivibriofibrisolvens*				
	*Clostridium bartlettii*				
	*Clostridium drakei*				
	*Eubacterium rectale*				
	*Megamonas hypermegale*				
	*Parabacteroides distasonis*				
	… for more see [57]				

## Abbreviations

Trp	tryptophan
Kyn	kynurenine
Kna	kynurenic acid
IDO	indoleamine 2,3-dioxygenase
TDO	tryptophan 2,3-dioxygenase
TPH	tryptophan hydroxylase
NAD	nicotinamide adenine dinucleotide
TMO	tryptophan 2-monooxygenase
TrD	tryptophan decarboxylase
ArAT	aromatic amino acid aminotransferase
TNA	tryptophanase
IAA	indole-3-acetic acid
IPA	indole-3-propionic acid
ILA	indole-3-lactic acid
IAAId	indole-3-acetaldehyde
IPA	indole-3-propionic acid
AhR	aryl hydrocarbon receptor
I3C	indole-3-carbinole
ISA	indole sulfuric acid
ROS	reactive oxygen species
IPYA	indole-3-pyruvate
IA	indole-acrylic acid
IAld	indole-3-aldehyde
I3A	indole-3-acetate
